# Manual for clinical language tractography

**DOI:** 10.1007/s00701-019-03899-0

**Published:** 2019-04-19

**Authors:** Lucius Fekonja, Ziqian Wang, Ina Bährend, Tizian Rosenstock, Judith Rösler, Lara Wallmeroth, Peter Vajkoczy, Thomas Picht

**Affiliations:** 1 Department of Neurosurgery, Charité - Universitätsmedizin Berlin, Berlin, Germany; 2 Cluster of Excellence: “Matters of Activity. Image Space Material”, Humboldt University, Berlin, Germany

**Keywords:** Brain tumor surgery, Diffusion tensor imaging, Language, Tractography, Neuroanatomy

## Abstract

**Background:**

We introduce a user-friendly, standardized protocol for tractography of the major language fiber bundles.

**Method:**

The introduced method uses dMRI images for tractography whereas the ROI definition is based on structural T1 MPRAGE MRI templates, without normalization to MNI space. ROIs for five language-relevant fiber bundles were visualized on an axial, coronal, or sagittal view of T1 MPRAGE images. The ROIs were defined based upon the tracts’ obligatory pathways, derived from literature and own experiences in peritumoral tractography.

**Results:**

The resulting guideline was evaluated for each fiber bundle in ten healthy subjects and ten patients by one expert and three raters.

Overall, 300 ROIs were evaluated and compared. The targeted language fiber bundles could be tracked in 88% of the ROI pairs, based on the raters’ result blinded ROI placements. The evaluation indicated that the precision of the ROIs did not relate to the varying experience of the raters.

**Conclusions:**

Our guideline introduces a standardized language tractography method for routine preoperative workup and for research contexts. The ROI placement guideline based on easy-to-identify anatomical landmarks proved to be user-friendly and accurate, also in inexperienced test persons.

## Introduction

We propose a user-friendly protocol for tractography of the major language fiber bundles. The ROI definition is based on structural T1 MPRAGE MRI images. The approach to define ROIs on high-resolution structural images instead of dMRI-based FA images allows standardized presurgical tractography of white matter tracts by delineating their obligatory pathways. In brain tumor patients, the white matter tracts can either be displaced, disrupted, infiltrated, or changed otherwise by pathology. A dMRI is a visually diffuse image dataset which provides an insufficient template to set ROIs for fiber tractography in relation to cerebral structures, especially in patients with brain tumors. Therefore, we propose an approach in which the ROI placement is guided by anatomical landmarks. This approach allows specific delineation of the individual white matter anatomy, even in the case of tumorous soft tissue displacement and infiltration. Our principal intention is to provide a user-friendly guideline and ROI standard that facilitates tractography of the major language fiber bundles, which can be used in a clinical setting.

The importance of achieving maximal tumor resection while preserving essential brain functions has been stressed in recent studies. Yet, studies on tractography of language-related fiber bundles as a tool for preoperative planning have been sparse and provide variable methodologies for ROI placements. Publications on language tractography have focused on consensus protocols in normalized space or on the functionality of the white matter tracts [7, 11, 15, 25, 34, 38]. However, those publications have not provided standard approaches on how to obtain reliable clinical language tractography results, i.e., in pathological brains, guided by cerebral structures or without the need for normalization [4, 36, 43]. Studies proposing the use of noninvasive cortical mapping results to place ROIs, which are linked with language function, suffer from the limited validation of the applied technology [9, 32, 33].

## Materials and methods

T1 MPRAGE MRIs were registered with dMRIs. The ROIs for the language-relevant fiber bundles are visualized on an axial, coronal, or sagittal view of T1 MPRAGE images. We defined the ROIs based upon the tracts’ obligatory pathways, derived from literature and own experiences in peritumoral tractography. The resulting guideline was evaluated in HCP and clinical datasets with different qualities. We established the ROI protocol on a cohort of ten healthy HCP subjects and tested it in clinically derived datasets. The clinically derived datasets included five myelopathy patients without supratentorial tumors and five patients with left perisylvian tumors.

## Data acquisition

For healthy average subjects, HCP data was acquired from the S1200 release.

Data acquisition for the HCP has been described in detail elsewhere [42].

See also http://humanconnectome.org/about/consortium-publications.php and http://www.humanconnectome.org/documentation/citations.html for updated lists of HCP publications and further information.

Both, for patients without cerebral tumors and patients with tumors in language-eloquent areas, data was acquired at Charité University Hospital, Berlin, Department of Neuroradiology, on a 3-T Skyra scanner. Diffusion data included 2-mm isotropic resolution whole-brain acquisitions; TR/TE 7500/95 ms; 1 shell b-value = 1300 s/mm^2^ with 60 directions per shell. The scans were performed with a standard ep2d sequence, for an acquisition time of 5:47 min. The structural acquisitions included a T1 MPRAGE anatomical sequence 1 mm isotropic resolution; TR/TE 2300/2.32 ms; TI 900 ms, flip angle 8°, for an acquisition time of 5:18 min.

## Preprocessing and processing

For definition and evaluation of ROI feasibility, we used tractography applications of different complexity (MRtrix3 and Brainlab Elements) to ensure cross-software validity. For usage within MRtrix3, the dMRI data has been denoised, corrected for eddy currents and motion and underwent a bias field correction for tractography purposes [39, 41, 44]. Probabilistic tractography has been performed with the iFOD2 algorithm by using a seeding and an inclusion spherical ROI [40]. The iFOD2 is a probabilistic algorithm that takes CSD-estimated FOD fields as input. Tracking parameters were set to default with a FOD amplitude cutoff value of 0.1, a streamline minimum length of 5 × voxel size and a maximum streamline length of 100 × voxel size. Each fiber bundle consists of 5000 streamlines.

FSL’s FLIRT has been used for registration of structural T1 MPRAGE images with dMRI images [16, 20, 21]. For preprocessing and registration in Brainlab Elements, we applied the proprietary cranial distortion correction module. Brainlab Elements offers a deterministic FACT and TEND algorithm [30, 45]. For a refinement of specific delineations of the fiber bundles, we suggest to use exclusion ROIs to additionally guide the fiber tracking algorithm. Those exclusion ROIs are highly dependent on the individual anatomy and tractography results; we therefore do not provide specific exclusion regions.

## Theory for ROI placement guidelines

The proposed guideline for tractography of the major language fiber bundles is based on two assumptions: first, the so-called major language fiber bundles play an essential role in language function. They are defined based on the current literature. The fiber bundles of interest are AF, FAT, IFOF, ILF, and UF. Literature suggests that tumors and surrounding edema involving the frontal portion of the AF results in deficits in repetition and fluency. Combined functional MRI and tractography showed in patients with frontal lobe gliomas that aphasic symptoms are more frequent in posteriorly to classical Broca’s area located tumors. Conduction aphasia was observed in all patients with gliomas and interruption of the AF’s direct segment and relative exemption of the anterior part [1, 2]. The FAT connects the posterior Broca area with medial frontal areas including pre-SMA and cingulate cortex. Stimulations of SMA, pre-SMA, and anterior cingulate cortex have been described to produce both speech arrest and vocalization [3]. Patients with lesions in these areas suffer from reduced spontaneous speech production, stuttering, different degrees of speech impairment, flat and monotonous intonation, as well as dysfluency [22, 37]. The involvement of IFOF in language function is currently not thoroughly understood but has been demonstrated to include functions like reading and writing as well as other semantic aspects. Stimulation of IFOF is recognized to result in semantic paraphasia [10, 12, 27–29]. The ILF transfers visual information from occipital areas to the temporal lobe and is considered to matter in visual object recognition, semantic processing, and lexical-phonological retrieval [5, 31]. The UF contributes to lexical retrieval, semantic associations, and processing as well as aspects of object naming and is related to single-word comprehension [6, 17, 26]. Further information regarding functions and relevance of the abovementioned fiber bundles in the language network, derived from direct cortical stimulation, has been described in detail elsewhere [7, 10, 11, 13, 25, 38]. In addition to the five chosen fiber bundles (AF, FAT IFOF, ILF, UF), the MdLF or even more tracts might be implemented in the future as another distinct part of the language network. Moreover, all fiber bundles have obligatory pathways, which are as well in pathological brains stable in their spatial relation to certain anatomical landmarks. Second, the refinement of MRI technology over the recent years provides high-resolution structural imaging also in the clinical routine. Therefore, identification of anatomical landmarks has become more straightforward and easier. These two assumptions lay the theoretical foundation for the proposed guideline of a high-quality, yet user-friendly and robust language fiber bundle tracking method. Our protocol intends to provide visual material as an easy-to-use guideline.

## ROI placement guidelines

For each fiber bundle, we suggest a two-ROI method, one seed and one target region for the tractography algorithm, delineated in an axial, coronal, or sagittal view. Additionally, if the fiber bundle of interest is difficult to delineate on the pathological hemisphere, it is advisable to delineate the fiber bundle first on the healthy hemisphere for introductory information.

### AF (Figs. 1a, b and 6)

The sagittal view is used as the main guiding view.

1. Find the section through the insula, where the temporal lobe is divided into the superior and inferior gyrus; the middle temporal gyrus might be not visible.
2. Set a seed ROI in the white matter region underneath the deepest point of the central sulcus, superior to the circular sulcus of insula.
3. Go to a slightly more lateral section through the insula towards the stem of the temporal lobe. Place a target ROI in the white matter at the level of the parieto-occipital sulcus, where the white matter changes its direction and forms the stem of the temporal lobe.
4. The posterior circular sulcus of insula may also be used as a landmark for the anterior region of the target ROI.

**Fig. 1:**
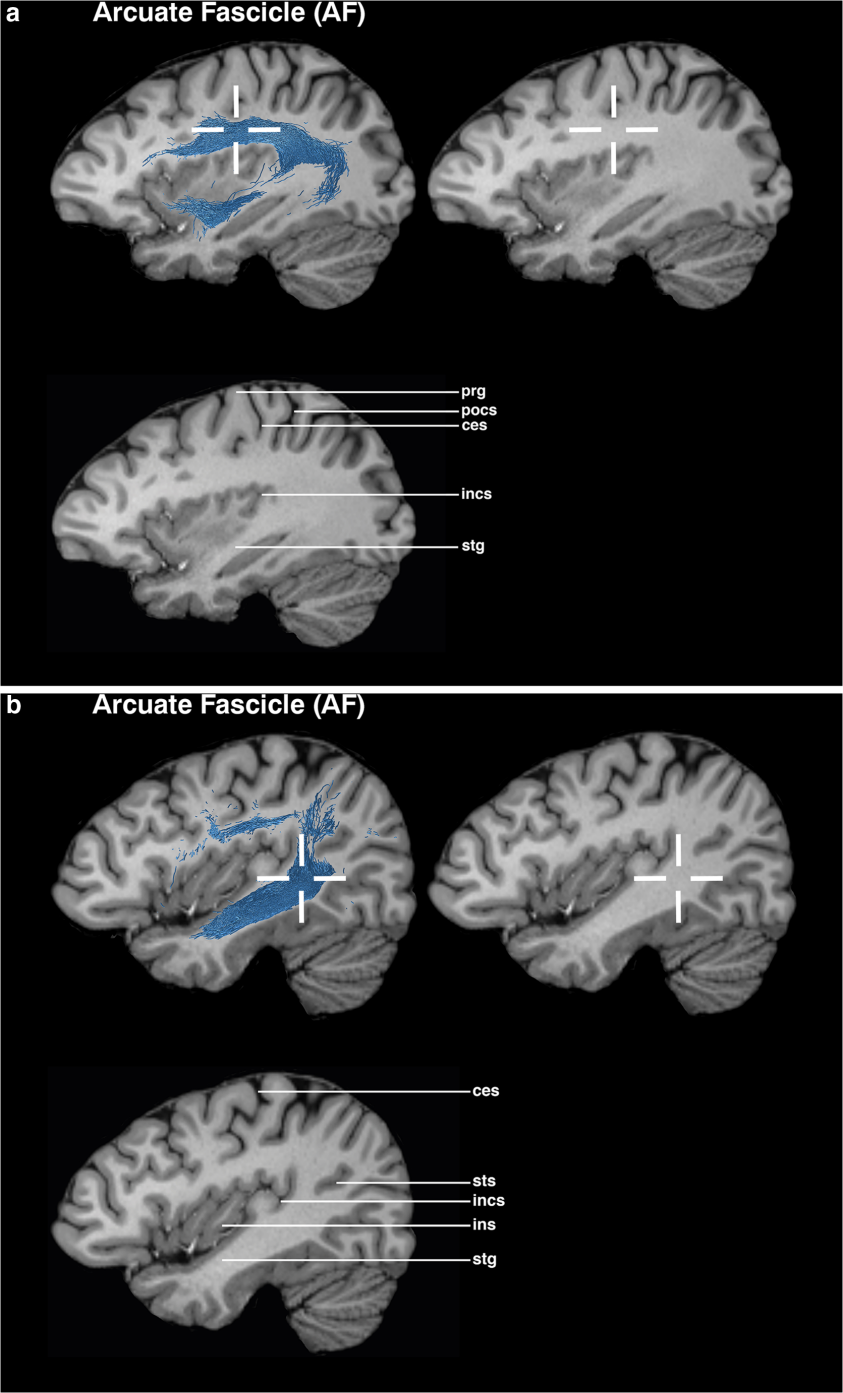
Visualizations for ROI placements for AF. Coronal, sagittal, or axial views of ROI placements with tractogram result. Top left shows target region with fiber tractography, top right shows target region without fiber tractography, and bottom left shows anatomical key structures for guidance

### FAT (SMA-subsegment) (Figs. 2 and 6)

The coronal view is used as the main guiding view.

1. Go to the section through the infundibulum and the amygdaloid body.
2. Set a seed ROI in the inferior-medial region of the superior frontal gyrus at the level of the deepest points of the cingulate sulcus and precentral sulcus.
3. The target ROI is placed in the white matter region, at the beginning of the inferior frontal gyrus region, at the deepest point of the circular sulcus of insula.

**Fig. 2:**
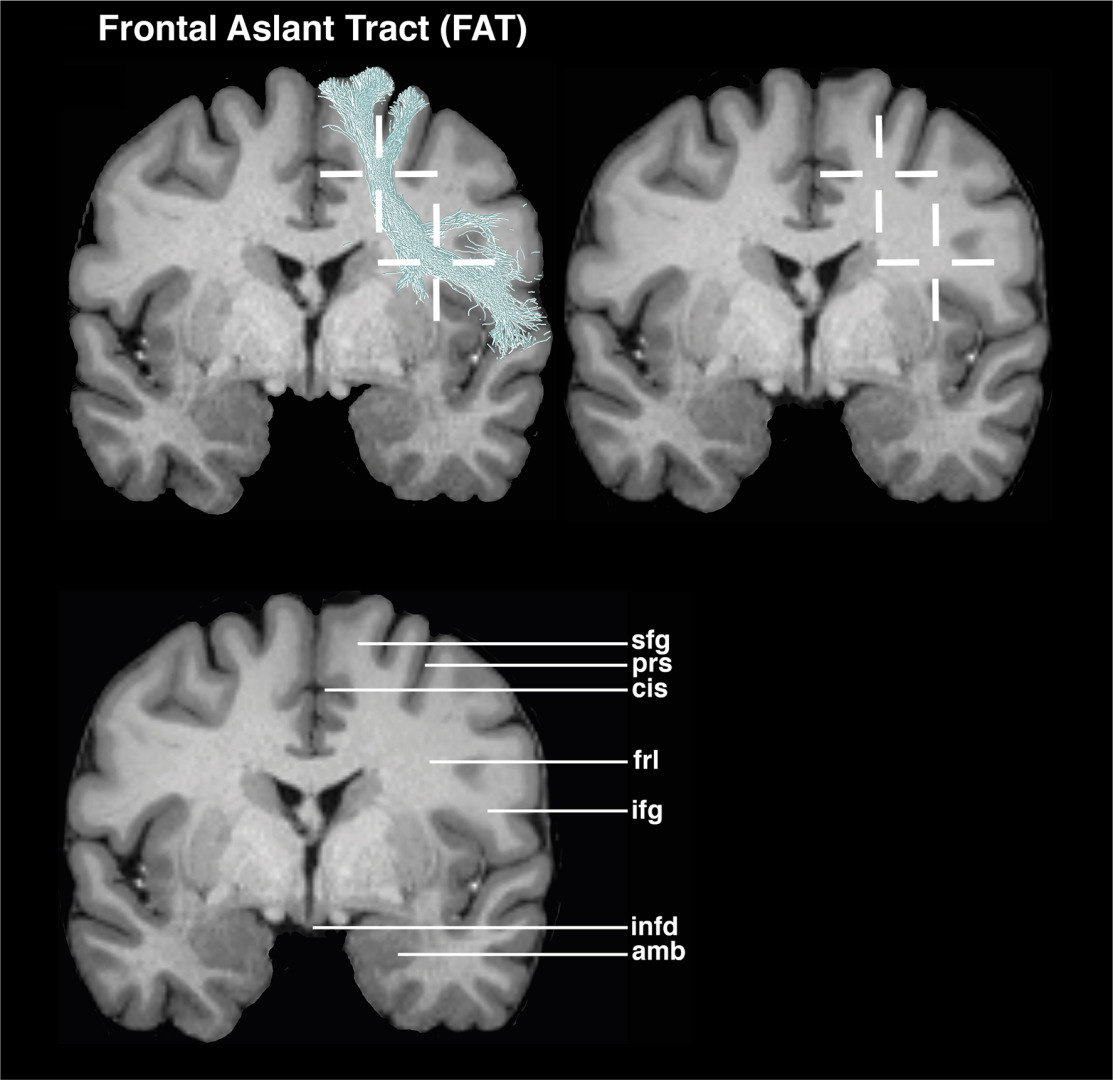
Visualizations for ROI placements for FAT. Coronal, sagittal, or axial views of ROI placements with tractogram result. Top left shows target region with fiber tractography, top right shows target region without fiber tractography, and bottom left shows anatomical key structures for guidance

### IFOF (Figs. 3 and 6)

The axial view is used as the main guiding view.

1. Go to the section through the head of caudate nucleus, circular sulcus of insula and superior colliculus. Place a seed ROI in the white matter region between the genu of corpus callosum and the anterior part of the head of caudate nucleus.
2. Place a target ROI in the white matter region of the middle temporal gyrus, anterior to the radiation of corpus callosum.

**Fig. 3:**
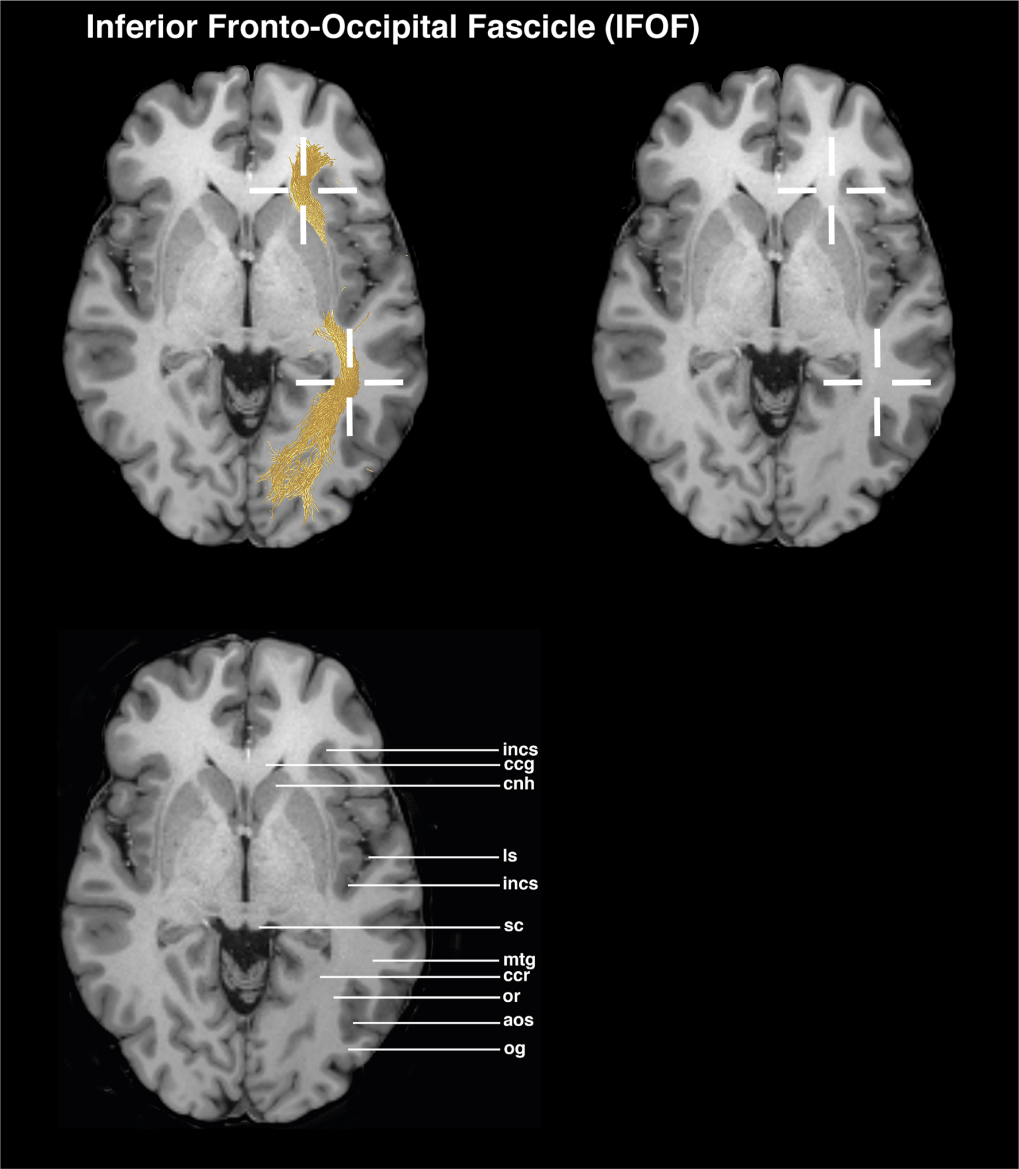
Visualizations for ROI placements for IFOF. Coronal, sagittal, or axial views of ROI placements with tractogram result. Top left shows target region with fiber tractography, top right shows target region without fiber tractography, and bottom left shows anatomical key structures for guidance

To exclude the corpus callosum tracts, the ROIs should be placed in between the area of the genu of corpus callosum and the splenium of corpus callosum.

In addition, the ROIs should be set anterior to the calcarine sulcus and striate area.

The location of the target ROI for the IFOF neglects putative projection of these bundles in the parietal cortex [8, 18].

### ILF (Figs. 4a, b and 6)

The axial view is used as the main guiding view.

1. Go to the section through the minor forceps and the inferior colliculus.
2. Set a seed ROI in the temporal lobe in the white matter region of the superior temporal gyrus, between the deepest point of the lateral sulcus and the deepest point of the circular sulcus of insula.
3. Go to the section through the head of caudate nucleus and the genu and splenium of corpus callosum.
4. Place a target ROI in the white matter part of the occipital gyri, in the region of the deepest point of the anterior occipital sulcus, lateral to the parieto-occipital sulcus.

**Fig. 4:**
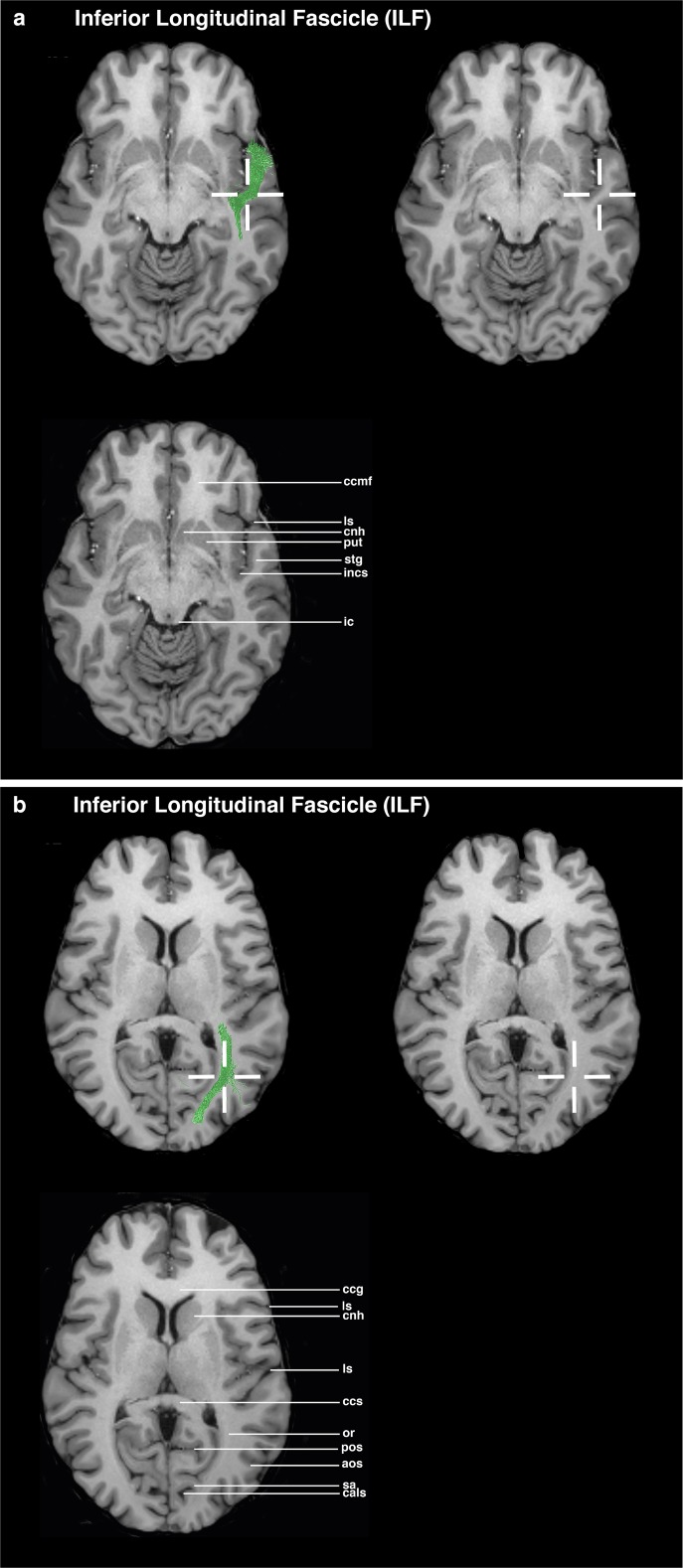
Visualizations for ROI placements for ILF. Coronal, sagittal, or axial views of ROI placements with tractogram result. Top left shows target region with fiber tractography, top right shows target region without fiber tractography, and bottom left shows anatomical key structures for guidance

### UF (Figs. 5a, b and 6)

The axial view is used as the main guiding view.

1. Go to the section through the temporal lobe, cerebral peduncle, and optic chiasm.
2. Set a seed ROI in the anterior part of the temporal lobe’s white matter, in the region of the deepest point of the superior temporal sulcus.
3. Go to the section through the minor forceps and the inferior colliculus.
4. Set a target ROI between the circular sulcus of insula and the anterior parts of the putamen and the head of caudate nucleus.

**Fig. 5:**
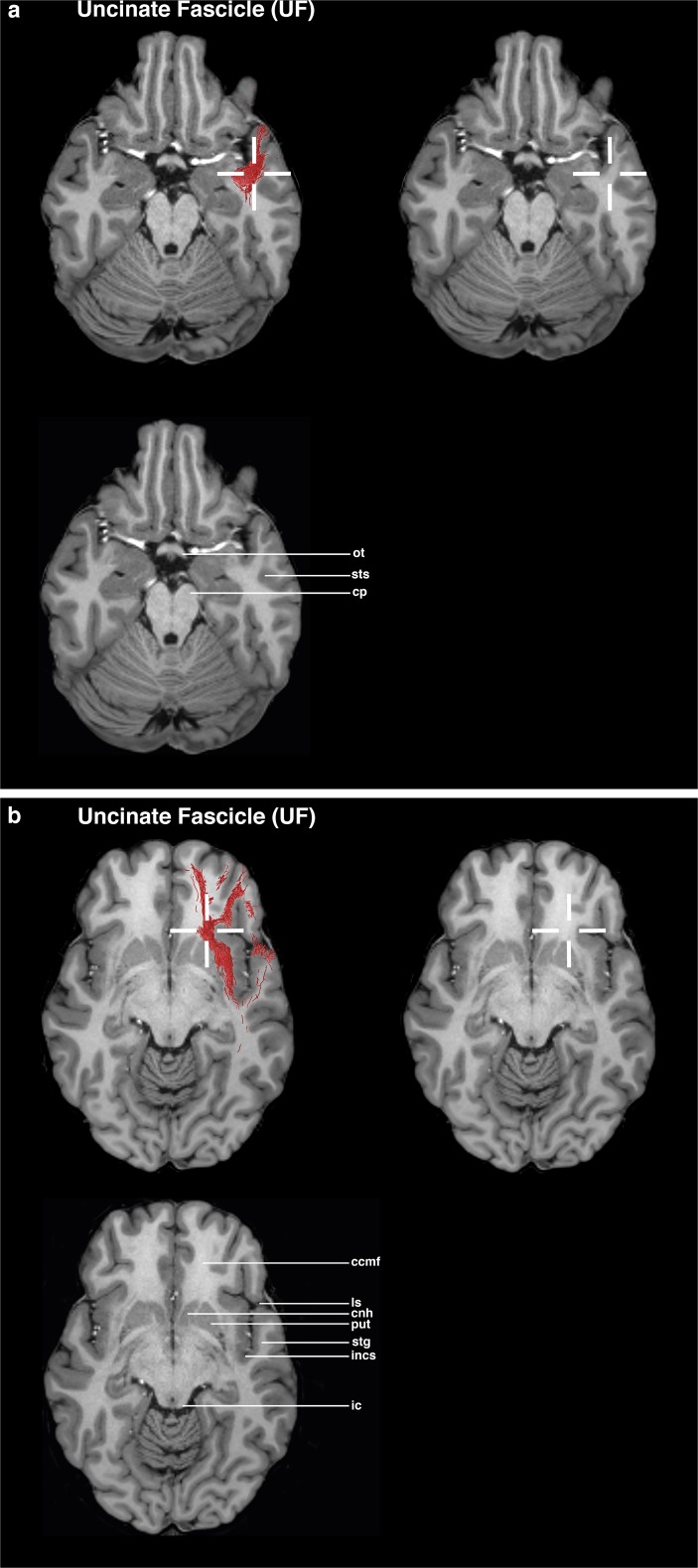
Visualizations for ROI placements for UF. Coronal, sagittal or axial views of ROI placements with tractogram result. Top left shows target region with fiber tractography, top right shows target region without fiber tractography, and bottom left shows anatomical key structures for guidance

**Fig. 6:**
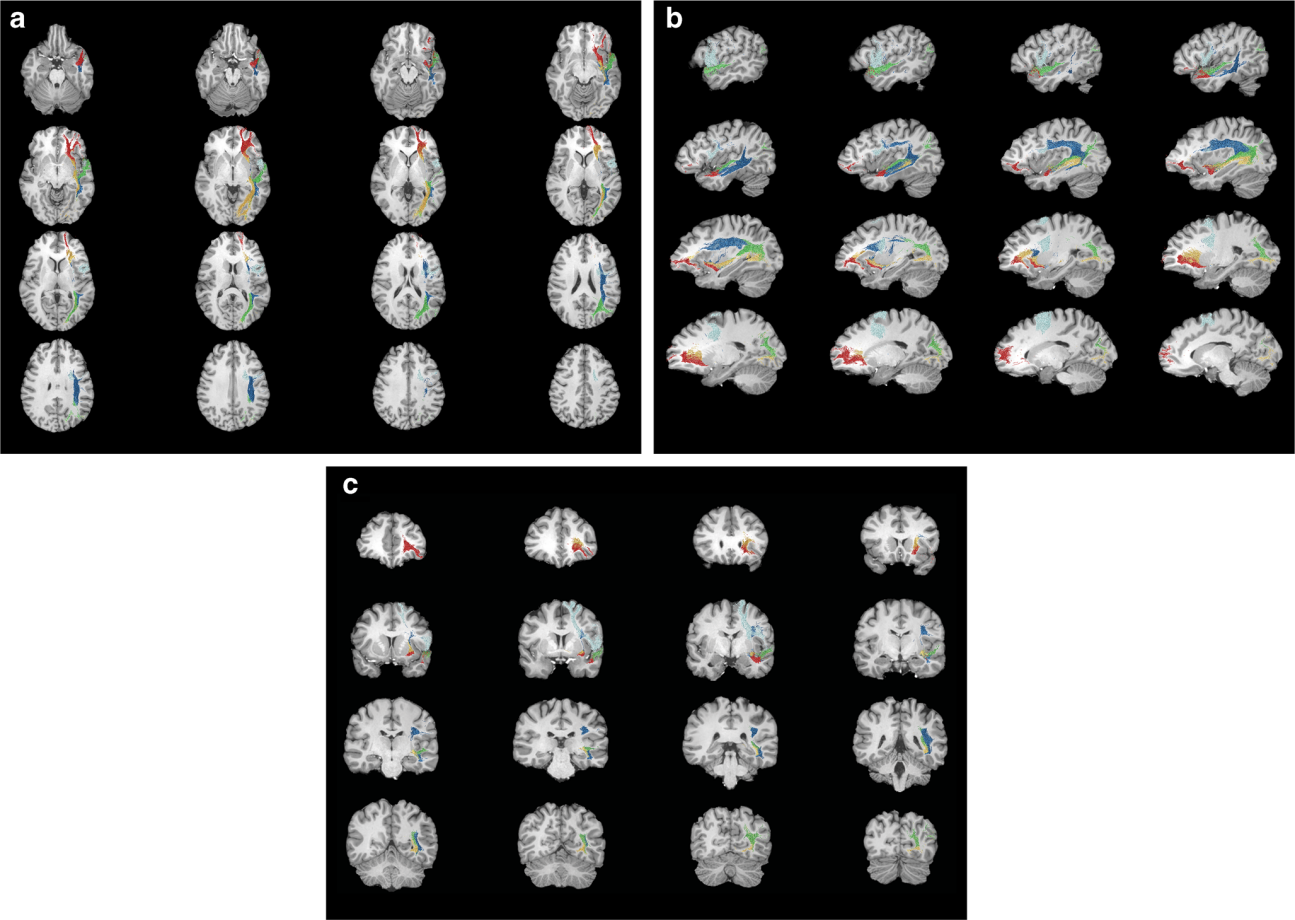
MRI-based lightbox overview with probabilistic fiber tractography results of the five major language fiber bundles. **a** Axial view. **b** Sagittal view. **c** Coronal view

## Evaluation

The proposed ROI placement protocol was established in ten HCP subjects and tested in five myelopathy patients without supratentorial tumors and in five patients with left perisylvian tumors. The testing examined the feasibility of the ROIs. After establishing the ROI placement guideline, an evaluation by three neurosurgeons with different expertise levels (1, 2, and 4 years) concerning neuroanatomical and tractography knowledge was conducted. The raters were instructed to mark the ROIs on T1 MPRAGE MRI images in the Brainlab application, based on the proposed ROI guidelines. The raters were not allowed to perform tractography; thereby, the raters had no possibility to check the feasibility of their ROIs. All raters placed two ROIs per fiber bundle in the left hemisphere, resulting in 300 ROI placements. The evaluation of the ROI placement was performed by analyzing the ROI positioning of three raters. The evaluation focused on the precision of the ROIs being either inside or outside the target ROI, defined as a 6-mm radius. The center is located within the crosshairs in Figs. 1, 2, 3, 4, and 5. The precision of the ROIs, based on the abovementioned delineation and figures, was measured with the Brainlab Elements program by measuring the distances to the author’s suggested ROI circumference (Figs. 1, 2, 3, 4, and 5). ROIs which had been placed more outside the circumference of the target ROI were defined as misplacements. If the ROI was misplaced, but touched the fiber bundle’s obligatory pathway, tractography could still be performed. The tractography outcome was evaluated on basis of the visual results by using the ROIs as seeding and inclusion areas. Tract feasibility and potential white matter tract displacement by pathology were assessed and compared to tractography results from literature [7, 14, 19, 46].

## Results

Overall, 300 ROIs were evaluated and compared. The targeted fiber bundles could be tracked via 132 out of the 150 ROI pairs, based on the blinded ROI placements (88% overall success). Tractography of 16 (16/150) fiber bundles was not possible, caused by at least one ROI being placed outside the target area (10.7%). ROIs for 76 fiber bundles were placed precisely (50.7%). In 58 cases, the fiber bundles could be tracked although the ROIs were not placed in the target area, but still touched the fiber bundles’ pathways (38.7%). The fiber tracking parameters in Brainlab Elements were 12-mm-diameter ROIs, minimum track length of 50 mm, FA cutoff value of 0.15, and maximum angulation value of 90°. Tractography was always successful in case both ROIs were placed in a 6-mm radius of the suggested ROI’s center or placed along the fiber bundles’ obligatory pathways. The evaluation indicated that the precision of the ROIs did not relate to the experience of the raters. The three raters misplaced ROIs for 4, 6, and 6 fiber bundles, so that those fiber bundles could not be tracked (92.0%, 88.0%, and 88.0% individual rater success rate). Misplacements occurred more often in tumor patients. Tractography of 16 fiber bundles was not possible in the tumor patient group, respectively, of two fiber bundles in the myelopathy patient group (78.7% and 97.3% overall success). In the tumor patient group, tractography of following fiber bundles was not possible: AF in four different patients (5× in the tumor group, 66.7% success rate in the tumor group and 83.3% success rate overall), FAT in one patient (1× in the tumor group, 93.3% success rate in the tumor group and 96.7% success rate overall), IFOF in three different patients (4× in the tumor group, 1× in the myelopathy group, 73.3% success rate in the tumor group, 93.3% success rate in the myelopathy group, and 83.3% success rate overall), ILF in one patient (1× in the tumor group, 93.3% success rate in the tumor group and 96.7% success rate overall), and UF in four different patients (5× in the tumor group, 1× in the myelopathy group, 66.7% success rate in the tumor group, 93.3% success rate in the myelopathy group, and 80.0% success rate overall). In one tumor patient, tracking of the UF was not possible by the raters’ ROIs. In this case, the tumor specifically infiltrated the UF pathway (Table 1). The standard deviation and coefficient of variation values are shown in Table 1. Fleiss’ kappa was used to assess differences in performances of ROI placements among the three raters which resulted in an inter-rater reliability of κ = 0.300.

**Table 1:** Table showing the raters’ ROI placements

		Tumor	Rater	AF		FAT		IFOF		ILF		UF	
Case no.	Sex, age	Location (side, lobe)		ROI1 (mm)	ROI2 (mm)	ROI1 (mm)	ROI2 (mm)	ROI1 (mm)	ROI2 (mm)	ROI1 (mm)	ROI2 (mm)	ROI1 (mm)	ROI2 (mm)
1	F, 36	–	I	–	2.0s	–	–	–	22.6a	–	–	–	14.6p
			II	–	–	–	–	–	19.8a	–	–	–	–
			III	–	–	–	–	–	–	–	–	–	–
2	M, 47	–	I	–	2.0s	–	–	–	–	–	17.2a	–	–
			II	–	–	–	–	–	–	–	–	–	12.2p
			III	7.9il	–	–	11.1l	–	–	10.4ip	–	–	2.0p
3	M, 47	–	I	6.3l	9.6s	6.4sl	18.6il	–	–	–	–	–	–
			II	–	8.8i	9.8il	–	–	7.2i	17.0p	6.2si	–	3.6a
			III	–	–	3.2p	3.2p	–	22.4p	–	–	–	–
4	M, 52	–	I	–	6.9m	13.1l	–	–	–	8.1sa	–	10.3ai*	11.4pi*
			II	3.5l	7.5a	–	–	5.6l*	–*	23.2a	–	7.3p	–
			III	9.1l	–	–	–	–	–	–	5.8s	6.0l	–
5	F, 55	–	I	–	10.4s	–	–	–	–	–	–	–	–
			II	–	21.6s	–	–	–	13.3a	22.0pm	6.6s	3.8p	–
			III	–	8.9p	–	–	–	–	–	–	–	–
6	F, 33	L, frontal	I	10.5a	7.2s	–	–	–	13.7a	–	13.1a	–	–
			II	5.9p	12.4i	–	–	–	–	10.2p	–	–	–
			III	13.9p*	26.0p*	–	–	–	5.67s	–	–	–	–
7	F, 34	L, temporal	I	–	–	–	–	14.1s	18.9a	–	–	–*	18.1a*
			II	6.7a*	20.8i*	–	–	–*	28.1a*	6.7p	6.9l	–	–
			III	13.0p	12.0p	–	–	–*	28.5a*	–	–	–	–
8	F, 31	L, frontal	I	–*	–*	–	–	7.3*	–*	–	–	–	–
			II	–	23.2i	–	–	–*	10.2s*	–	–	–*	10.7ip*
			III	3.6l	2.0a	–*	–*	–	–	–	–	–	–
9	F, 53	L, frontal	I	–	–	–	–	–	–	–	–	21.3a	14.3p
			II	–	–	–	–	–	–	15.3pm	–	5.6p	–
			III	–	7.9ip	–	6.7l	–	11.8a	11.1p	–	–	–
10	F, 65	R, temporal	I	10.3il*	–*	–	–	5.1s	9.0p	26.4a*	–*	24.6a*	15.5im*
			II	–	3.4l	–	–	–	18.6p	–	–	3.6s*	7.0sm*
			III	10.0a*	10.0i*	16.1lp	6.4p	9.0i	12.9ai	–	–	10.5i*	8.1ip*
Mean				3.4	6.8	1.6	1.5	1.4	8.1	5.0	1.9	3.1	3.9
Standard deviation				4.7	7.7	4.1	4.1	3.4	9.6	8.2	4.3	6.3	6.0
Coefficient of variation				1.4	1.1	2.6	2.7	2.5	1.2	1.6	2.3	2.0	1.5

*l* lateral, *i* inferior, *m* medial, *a* anterior, *s* superior

*Fiber bundles which were not traceable

## Discussion

Our guideline introduces a standardized language tractography method for routine preoperative workup as well as for research contexts. The main purpose was to develop a user-friendly guideline and ROI standard that eases tractography of the major language fiber bundles. Knowledge from basic science was subsumed, tested in tumor patients and made available for routine use by describing easy-to-identify anatomical landmarks for ROI placements. The raters’ amount of misplaced ROIs (4, 6, and 6) show that a correct placement of suggested ROIs is feasible by different expertise level and does not give advantage to more experienced users. The proposed ROI placement guideline let to successful delineation of the five major language fiber bundles in 78.7% in brain tumor patients and in 97.3% in myelopathy patients, even when performed by nonexperts and a beginner. If the ROIs were placed correctly, they always resulted in successful tractography in myelopathy patients (100%) and in tumor patients in nearly every case (94.7%). Inter-rater reliability for ROI placements (κ = 0.300) showed fair agreement according to Landis and Koch [23]. However, it should be noted that the value represents a comparison of three different competence levels. The increased difficulty of fiber tractography near brain tumors is mirrored in these results. This difficulty is mainly caused by impaired structural white matter integrity and less by the difficulty to identify the proposed anatomical landmarks [19].

The ROI placement was purely anatomically, i.e., the subjects were not allowed to test their ROI feasibilities by performing fiber tractography. Additional adjustments of the ROIs allows for an even higher success rate. Initially, the research software MRtrix3 had been used to define and test the ROIs before evaluating them with the clinical software Brainlab Elements. In the more complex tractography software MRtrix3, the program’s standard settings of the iFOD2, with the same spherical ROI sizes provided good fiber tractography results. Furthermore, deterministic tractography can never account for uncertainty or distributed connectivity in white matter tracts (https://www.humanconnectome.org/study/hcp-young-adult/project-protocol/diffusion-tractography). From a methodological point of view, it has to be stressed that tractography suffers from a number of limitations such as tracking is terminated by FA or FOD thresholds, which does not mean that there are no fibers. This needs to be taken into account for surgical planning. The FA values provide a measure which may be the sum of multiple structures and factors within a voxel. Due to this fact, the FA values have to be interpreted in relation to anatomy, pathology, increase in extracellular or intracellular water, etc. Furthermore, the FA values have to be interpreted in a critical fashion, especially with clinical data, where voxel sizes are usually bigger than in experimental research settings due to scan time considerations [24, 35].

## Limitations

The proposed guideline is based on the existing state-of-the-art literature and has been applied and tested by members of our institution. To evaluate the proposed ROIs and implement them in a clinical setting, multiple computer programs with different fiber tractography algorithms were used. In this study, we had no intention to compare different tractography algorithms. The different algorithms were only used for feasibility testing of our proposed ROIs to enable tractography of the tracts’ obligatory pathways. Conclusions about the feasibility of the guideline need to be confirmed in further studies.

## Conclusion

The introduced method with high-resolution structural T1 MPRAGE images provides a precise definition of ROIs and provides orientation on detailed information concerning individual-specific anatomical structures and landmarks by usage of T1 MPRAGE images. With the ongoing evolvement of MRI technology resulting in high-resolution images, individual-specific anatomically defined ROIs for fiber tractography will become an even more user-friendly method in the foreseeable future with the potential for automation in clinical computer programs.

## Abbreviations

AF: Arcuate fasciculus
AMB: Amygdaloid body
ANG: Angular gyrus
AOS: Anterior occipital sulcus
AP: Anterior–posterior
CALS: Calcarine sulcus
CCG: Genu of corpus callosum
CCMF: Minor forceps of corpus callosum
CCR: Radiation of corpus callosum
CCS: Splenium of corpus callosum
CES: Central sulcus
CIS: Cingulate sulcus
CNH: Head of caudate nucleus
CP: Cerebral peduncle
CR: Corona radiata
CSD: Constrained spherical deconvolution
dMRI: Diffusion-weighted magnetic resonance imaging
FA: Fractional anisotropy
FAT: Frontal aslant tract
FOD: Fiber orientation distributions
FRL: Frontal lobe
FSL: FMRIB Software Library (FMRIB = [Oxford Centre for] Functional MRI of the Brain)
IC: Inferior colliculus
IFG: Inferior frontal gyrus
IFOF: Inferior fronto-occipital fasciculus
ILF: Inferior longitudinal fasciculus
IN: Insula
INCS: Circular sulcus of insula
INF: Infundibulum
HCP: Human connectome project
LS: Lateral sulcus
MB: Multiband
MdLF: Middle longitudinal fasciculus
MNI: Montreal Neurological Institute (standard brain)
MPRAGE: Magnetization-prepared rapid gradient-echo
MRI: Magnetic resonance imaging
MTG: Middle temporal gyrus
OG: Occipital gyri
OR: Optic radiation
OT: Optic tract
PA: Posterior-anterior
POCS: Posterior central sulcus
POG: Postcentral gyrus
POS: Parieto-occipital sulcus
PRG: Precentral gyrus
PRS: Precentral sulcus
PUT: Putamen
ROI: Region of interest
SA: Striate area
SC: Superior colliculus
SFG: Superior frontal gyrus
SMA: Supplementary motor area
SMG: Supramarginal gyrus
STG: Superior temporal gyrus
STS: Superior temporal sulcus
TI: Inversion time
TR: Repetition time
TE: Echo time
UF: Uncinate fasciculus
WU-Minn: Washington University - University of Minnesota

## References

[1] Bizzi A, Nava S, Ferre F (2012). Aphasia induced by gliomas growing in the ventrolateral frontal region: assessment with diffusion MR tractography, functional MR imaging and neuropsychology. Cortex.

[2] Catani M, Dell'acqua F, Bizzi A (2012). Beyond cortical localization in clinico-anatomical correlation. Cortex.

[3] Catani M, Dell'acqua F, Vergani F (2012). Short frontal lobe connections of the human brain. Cortex.

[4] Catani M, Howard RJ, Pajevic S (2002). Virtual in vivo interactive dissection of white matter fasciculi in the human brain. Neuroimage.

[5] Catani M, Jones DK, Donato R (2003). Occipito-temporal connections in the human brain. Brain.

[6] Catani M, Mesulam MM, Jakobsen E (2013). A novel frontal pathway underlies verbal fluency in primary progressive aphasia. Brain.

[7] Catani M, Thiebaut de Schotten M (2008). A diffusion tensor imaging tractography atlas for virtual in vivo dissections. Cortex.

[8] Caverzasi E, Papinutto N, Amirbekian B (2014). Q-ball of inferior fronto-occipital fasciculus and beyond. PLoS One.

[9] Cochereau J, Deverdun J, Herbet G (2016). Comparison between resting state fMRI networks and responsive cortical stimulations in glioma patients. Hum Brain Mapp.

[10] Duffau H (2008). The anatomo-functional connectivity of language revisited. New insights provided by electrostimulation and tractography. Neuropsychologia.

[11] Duffau H (2015). Stimulation mapping of white matter tracts to study brain functional connectivity. Nat Rev Neurol.

[12] Duffau H, Gatignol P, Mandonnet E (2005). New insights into the anatomo-functional connectivity of the semantic system: a study using cortico-subcortical electrostimulations. Brain.

[13] Duffau H, Lopes M, Arthuis F (2005). Contribution of intraoperative electrical stimulations in surgery of low grade gliomas: a comparative study between two series without (1985-96) and with (1996-2003) functional mapping in the same institution. J Neurol Neurosurg Psychiatry.

[14] Field AS, Alexander AL, Wu YC (2004). Diffusion tensor eigenvector directional color imaging patterns in the evaluation of cerebral white matter tracts altered by tumor. J Magn Reson Imaging.

[15] Friederici AD (2011). The brain basis of language processing: from structure to function. Physiol Rev.

[16] Greve DN, Fischl B (2009). Accurate and robust brain image alignment using boundary-based registration. Neuroimage.

[17] Grossman M, McMillan C, Moore P (2004). What’s in a name: voxel-based morphometric analyses of MRI and naming difficulty in Alzheimer’s disease, frontotemporal dementia and corticobasal degeneration. Brain.

[18] Hau J, Sarubbo S, Perchey G (2016). Cortical terminations of the inferior fronto-occipital and uncinate fasciculi: anatomical stem-based virtual dissection. Front Neuroanat.

[19] Jellison BJ, Field AS, Medow J (2004). Diffusion tensor imaging of cerebral white matter: a pictorial review of physics, fiber tract anatomy, and tumor imaging patterns. AJNR Am J Neuroradiol.

[20] Jenkinson M, Bannister P, Brady M (2002). Improved optimization for the robust and accurate linear registration and motion correction of brain images. Neuroimage.

[21] Jenkinson M, Smith S (2001). A global optimisation method for robust affine registration of brain images. Med Image Anal.

[22] Jurgens U, von Cramon D (1982). On the role of the anterior cingulate cortex in phonation: a case report. Brain Lang.

[23] Landis JR, Koch GG (1977). The measurement of observer agreement for categorical data. Biometrics.

[24] Lanfermann H, Schindler C, Jordan J (2015). Pharmacological MRI (phMRI) of the human central nervous system. Clin Neuroradiol.

[25] Leclercq D, Duffau H, Delmaire C (2010). Comparison of diffusion tensor imaging tractography of language tracts and intraoperative subcortical stimulations. J Neurosurg.

[26] Lu LH, Crosson B, Nadeau SE (2002). Category-specific naming deficits for objects and actions: semantic attribute and grammatical role hypotheses. Neuropsychologia.

[27] Makris N, Kennedy DN, McInerney S (2005). Segmentation of subcomponents within the superior longitudinal fascicle in humans: a quantitative, in vivo, DT-MRI study. Cereb Cortex.

[28] Mandonnet E, Nouet A, Gatignol P (2007). Does the left inferior longitudinal fasciculus play a role in language? A brain stimulation study. Brain.

[29] Martino J, Brogna C, Robles SG (2010). Anatomic dissection of the inferior fronto-occipital fasciculus revisited in the lights of brain stimulation data. Cortex.

[30] Mori S, Crain BJ, Chacko VP (1999). Three-dimensional tracking of axonal projections in the brain by magnetic resonance imaging. Ann Neurol.

[31] Mummery CJ, Patterson K, Wise RJ (1999). Disrupted temporal lobe connections in semantic dementia. Brain.

[32] Negwer C, Ille S, Hauck T (2017). Visualization of subcortical language pathways by diffusion tensor imaging fiber tracking based on rTMS language mapping. Brain Imaging Behav.

[33] Negwer C, Sollmann N, Ille S (2017). Language pathway tracking: comparing nTMS-based DTI fiber tracking with a cubic ROIs-based protocol. J Neurosurg.

[34] Picht T (2015). Navigated transcranial magnetic stimulation for preoperative mapping of the eloquent cortex. Nervenarzt.

[35] Qin YY, Li MW, Zhang S (2013). In vivo quantitative whole-brain diffusion tensor imaging analysis of APP/PS1 transgenic mice using voxel-based and atlas-based methods. Neuroradiology.

[36] Raffa G, Bahrend I, Schneider H (2016). A novel technique for region and linguistic specific nTMS-based DTI fiber tracking of language pathways in brain tumor patients. Front Neurosci.

[37] Rubens AB (1975). Aphasia with infarction in the territory of the anterior cerebral artery. Cortex.

[38] Sarubbo S, De Benedictis A, Merler S (2015). Towards a functional atlas of human white matter. Hum Brain Mapp.

[39] Smith SM, Jenkinson M, Woolrich MW (2004). Advances in functional and structural MR image analysis and implementation as FSL. Neuroimage.

[40] Tournier J-D, Calamante F, Connelly A (2010) Improved probabilistic streamlines tractography by 2nd order integration over fibre orientation distributions. Proceedings of the International Society for Magnetic Resonance in Medicine, 1670. In: International Society for Magnetic Resonance in Medicine, 2010

[41] Tustison NJ, Avants BB, Cook PA (2010). N4ITK: improved N3 bias correction. IEEE Trans Med Imaging.

[42] Van Essen DC, Ugurbil K, Auerbach E (2012). The Human Connectome Project: a data acquisition perspective. Neuroimage.

[43] Vassal F, Schneider F, Boutet C (2016). Combined DTI tractography and functional MRI study of the language connectome in healthy volunteers: extensive mapping of white matter fascicles and cortical activations. PLoS One.

[44] Veraart J, Novikov DS, Christiaens D (2016). Denoising of diffusion MRI using random matrix theory. Neuroimage.

[45] Weinstein D, Kindlmann G, Tensorlines LE (1999). Advection-diffusion based propagation through diffusion tensor fields. Proceedings of the conference on Visualization’99: celebrating ten years.

[46] Witwer BP, Moftakhar R, Hasan KM (2002). Diffusion-tensor imaging of white matter tracts in patients with cerebral neoplasm. J Neurosurg.

